# Improved characterization of the relationship between long intergenic non‐coding RNA Linc00152 and the occurrence and development of malignancies

**DOI:** 10.1002/cam4.2245

**Published:** 2019-07-04

**Authors:** Jiasheng Xu, Jingjing Guo, Yangkai Jiang, Yujun Liu, Kaili Liao, Zhonghua Fu, Zhenfang Xiong

**Affiliations:** ^1^ Department of Pathology The First Affiliated Hospital of Nanchang University Nanchang China; ^2^ Queen Mary College of Nanchang University Nanchang China; ^3^ Department of Clinical Laboratory The Second Affiliated Hospital of Nanchang University Nanchang China; ^4^ Department of Burns The First Affiliated Hospital of Nanchang University Nanchang China

**Keywords:** linc00152, long non‐coding RNA, malignant tumor

## Abstract

Linc00152, located on chromosome 2p11.2, is a long intergenic non‐coding RNA molecule with 828 nucleotides that is highly expressed in many types of human tumor tissues, especially in malignant tumors of the digestive system. Linc00152 promotes the occurrence and development of tumors by increasing tumor cell proliferation, invasion, metastasis, and apoptosis. Additionally, linc00152 contributes to the carcinogenesis of several cancers, including gastric cancer, liver cancer, hepatocellular carcinoma, gallbladder cancer, clear cell renal cell carcinoma, and colorectal cancer, by disturbing various signaling pathways (eg PI3K/AKT, mTOR, IL‐1, and NOTCH 1 signaling pathways). High linc00152 expression levels are associated with chemoresistance as well as poor prognosis and shorter survival. Continual advances made in the relevant research have indicated that linc00152 may be useful as a new tumor molecular biomarker, applicable for tumor diagnosis, targeted therapy, and prognosis assessment. This review summarizes the progress in the research into the relationship between linc00152 and the occurrence and development of malignancies based on molecular functions, regulatory mechanisms, and clinical applications.

## 1 INTRODUCTION

Long noncoding RNAs (lncRNAs), which comprise a group of RNA molecules with more than 200 nucleotides, were originally considered to be transcript “noise” because they do not directly encode proteins.^1^ Further research revealed that lncRNAs regulate the expression of target genes related to cell growth and development at various levels (eg epigenetic, transcriptional, and posttranscriptional regulation) via diverse methods, including through gene signature changes, chromatin modification, transcriptional activation and interference, and nuclear transport.^2^ Additionally, lncRNAs are more tissue‐specific than protein‐coding RNAs, with expression levels that not only vary from tissue to tissue, but also change in different parts of the same tissue.^3^, ^4^, ^5^, ^6^ Moreover, lncRNA expression exhibits strong temporal and spatial specificity, and the expression of the same lncRNA differs considerably during different developmental stages in the same tissue or organ.^7^, ^8^, ^9^, ^10^ Abnormal lncRNA expression is closely related to many diseases, including cancer. Linc00152 is a class of lncRNA that is expressed uncontrollably in various tumor tissues, especially in malignant tumors of the digestive system, and is highly correlated with the formation, development, and metastasis of tumors.^11^, ^12^ This review summarizes the progress in the research regarding the relationship between linc00152 and the occurrence and development of malignancies.

## 2 OVERVIEW OF LINC00152

Although lncRNAs are distributed in the cytoplasm, nucleus, and organelles, they are mainly present in the nucleus.^13^ At least 4%‐9% of mammalian genomes are eventually transcribed into lncRNAs.^14^ The secondary and tertiary structures of lncRNAs are complex and versatile, but there is currently no effective classification method available. According to the location and affinity of lncRNAs and the adjacent protein‐coding genes, lncRNAs can be classified into the following five categories^15^: (a) antisense lncRNAs, which are transcribed in the reverse direction from the adjacent coding genes and overlap other protein‐coding genes (eg shared exons); (b) intragenic lncRNAs (intronic lncRNAs), which originate from the intron of a protein‐coding gene but have a limited nonoverlapping region and are transcribed from relatively adjacent protein‐coding genes in either direction; (c) heterologous lncRNAs (divergent lncRNAs), which are transcribed in the opposite direction from the promoters of adjacent protein‐coding genes; transcription often involves several hundred base pairs from the transcription start site of the adjacent protein‐coding genes; (d) intergenic lncRNAs (lincRNAs), which are co‐transcribed from protein‐coding genes that do not share promoters, exons, or introns; and (e) enhancer‐type lncRNAs (enhancer RNAs), which are transcribed by enhancers and can regulate the long‐distance or short‐distance mediation of the interaction between an lncRNA enhancer and other transcription factors in the genome. Additionally, lincRNAs, which represent the main lncRNAs, are involved in many biological processes,^16^ such as cell cycle regulation, immune surveillance, and pluripotency of embryonic stem cells. More than 3,300 lincRNAs have been detected in the human genome. For example, linc00152, which comprises 828 nucleotides, is located on chromosome 2p11.2 and is transcribed from a region between 2 protein‐coding genes. Studies have revealed that linc00152 functions primarily as a molecular indicator that helps regulate the transcription of downstream genes. Moreover, linc00152 can affect the epidermal growth factor receptor (EGFR), phosphatidylinositol 3‐kinase (PI3K)/AKT,^17^, ^18^, ^19^ and mechanistic target of rapamycin (mTOR)^20^ signaling pathways, which regulate the expression of related target genes. Linc00152 regulates the methylation of lysine 27 at the amino terminal of histone H3 by recruiting a histone lysine methyltransferase (eg enhancer of zeste homolog 2 [EZH2]), which modulates gene expression at the epigenetic level. Furthermore, as a competitive endogenous RNA (ceRNA), it competitively binds to microRNAs (miRNAs) to regulate the abundance of the protein encoded by a particular gene and helps regulate the biological behavior of cells. However, the available information regarding linc00152 remains relatively limited, and only a few functions have been confirmed. The specific molecular mechanisms associated with many tumors have not been elucidated.

## 3 MOLECULAR FUNCTIONS AND MECHANISMS OF LINC00152 IN HUMAN TUMORS

There has recently been considerable interest in linc00152 among oncologists. It is abnormally expressed in various malignancies, such as gastric cancer, liver cancer, colon cancer, clear cell renal cell carcinoma, and lung cancer (Table 1), and contributes to the occurrence and development of tumors. However, linc00152 exhibits diverse molecular functions in different human tumors.

**Table 1:** Linc00152‐related cancers and the associated mechanisms

Cancer type	Related molecules	linc0015 expression level	Signal pathway
Gastric cancer 29	P15, P21	high	PI3K/AKT
Liver cancer 11	AFP, adhesion molecule promoter	high	mTOR
Hepatocellular carcinoma 20	EpCAM	high	mTOR
Hepatitis B virus‐associated hepatocellular carcinoma 49	HBx	high	
Gallbladder cancer 20, 39	SP1, hypoxia‐inducible factor‐1α	high	PI3K/ AKT
Renal clear cell carcinoma 34, 53	P16, miR‐205, (EZH2), LSD1 and histone H3	high	/
Lung cancer 41, 45	EZH2, IL‐24	high	IL‐1
	P21	high	PI3K/AKT
Infantile Hemangiomas 37	/	high	/
Glioma 46, 47	MiR‐16, BMI1	high	
	miR‐103a‐3p, FEZF1, CDC25A	high	
Triple‐negative breast cancer 36	BRCA1/PTEN	high	
Colorectal cancer 50, 51, 52	FU, MIr‐139‐5p	high	NOTCH 1
	CK1		

### 3.1 Overexpression of linc00152 enhances cell proliferation, invasion, and metastasis, while inhibiting apoptosis

To date, high linc00152 levels have been detected in many cancers,^21^, ^22^, ^23^ and the associated mechanisms mediating tumorigenesis are complex and specific. Deregulated linc00152 expression has been observed in head and neck squamous cell carcinoma, in which five types of lncRNAs, including linc00152, are differentially expressed.^22^ A previous study by Chen et al 23 involving an analysis of 60 lung cancer tissues by RT‐PCR revealed that linc00152 is expressed significantly more in lung cancer tissue than in normal lung tissue.

Yu et al 24 confirmed that linc00152 is highly expressed in pancreatic ductal adenocarcinoma (PDAC), which is a very aggressive cancer with a poor prognosis and short overall survival. Their qRT‐PCR and transwell assay results indicated that the knockdown of linc00152 prevents PDAC cell proliferation and invasion, thereby verifying the oncogenic property of linc00152. The involvement of linc00152 in cell proliferation has also been confirmed in gastric cancer,^25^, ^26^, ^27^, ^28^, ^29^, ^30^, ^31^, ^32^ hepatocellular carcinoma (HCC),^11^, ^20^ pancreatic cancer,^33^ clear cell renal cell carcinoma,^34^ colorectal cancer,^35^ breast cancer,^36^ and infantile hemangioma.^37^

Cao et al 26 applied a cDNA microarray to analyze the profiles of differentially expressed lncRNAs in 88 gastric cancer tissue samples and paired adjacent normal tissues. They detected 80 kinds of differentially expressed lncRNAs, of which the expression levels of linc00152 were particularly significantly different. Subsequent research involving qRT‐PCR has confirmed that linc00152 is overexpressed in gastric cancer cells, and that the expression level is positively correlated with the invasiveness of tumor cells.^25^, ^26^, ^27^, ^28^, ^29^, ^30^, ^31^ After silencing linc00152 with small interfering RNA, the growth of gastric cancer cells reportedly slows down and the cell cycle is arrested. Additionally, there is an increase in apoptosis, but a decrease in cell invasion and metastasis. Linc00152 expression is also upregulated in the gastric juice of patients.^27^ Another study revealed that cytoplasmic linc00152 expression is upregulated in many gastric cancer samples, and the knockdown of linc00152 can inhibit tumor growth.^28^ The linc00152‐induced cell proliferation in gastric cancer proves that linc00152 can bind to EZH2, suppressing p15 and p21 to accelerate the cell cycle. The expression of linc00152 is also positively related to tumor invasion, lymph node metastasis, and cancer progression (according to the tumor–node–metastasis [TNM] stage).^29^ Linc00152 also induces glycolysis, which is a key feature of gastric cancer, by inhibiting miR‐139‐5p, to control the production of the AMP‐activated, alpha 1 catalytic subunit.^34^, ^38^

In addition to gastric cancer cells, Cai et al 39, 40 discovered that linc00152 is also overexpressed in gallbladder cancer cells, with similar activities during the progression of cancer. Both in vitro and in vivo experiments have demonstrated that the upregulation of linc00152 expression promotes the proliferation and metastasis of gallbladder cancer cells and the growth of mouse xenografts, while also repressing apoptosis. Transcription factor SP1, which is important for tumor growth and differentiation, can specifically recognize the linc00152 promoter region and activate the PI3K/AKT signaling pathway to promote the proliferation and differentiation of gallbladder carcinoma cells. In mice, the knockdown of linc00152 in gallbladder carcinoma cells significantly inhibits cell invasion and metastasis, and markedly reduces peritoneal metastasis, suggesting that linc00152 is crucial for promoting the development, progression, and metastasis of gallbladder carcinoma cells.^39^ Additional research has revealed that linc00152 can be used as a miRNA “sponge” that “adsorbs” miR‐138, which in turn upregulates the expression of the hypoxia‐inducible factor‐1α gene and promotes tumor cell invasion and metastasis.^41^ Therefore, linc00152 may represent a new target for the diagnosis and treatment of gallbladder cancer.

A similar mechanism was observed in non‐small cell lung cancer (NSCLC). Specifically, Zhang et al 41 determined the knockdown of linc00152 can inhibit the cell cycle (especially the G1 phase) to delay cell proliferation. This was revealed by the considerable decrease in EGFR, PI3K, and AKT activities and the epithelial intercellular transition via the suppression of fibronectin and vimentin downstream of the EGFR/PI3K/AKT signaling pathway to inhibit cell invasion and migration, in addition to an increase in P21 production to promote apoptosis.^41^, ^42^ This not only clarified the linc00152 mechanism related to the progression of NSCLC but also suggests that linc00152 is a potential therapeutic target. Several studies concluded that linc00152 expression is also upregulated in lung adenocarcinoma (LUAD) cells.^43^, ^44^, ^45^ Additionally, linc00152 is overexpressed only in patients with a poor prognosis. The inhibition of linc00152 expression in LUAD cells may decrease cell proliferation and colony formation.^45^ However, Feng demonstrated that the underlying mechanism may involve the binding of linc00152 to EZH2, which negatively regulates IL‐24 production, thereby inhibiting tumor cell apoptosis. The two mechanisms may be both associated with lung cancer progression, and these results imply that linc00152 may be a potential target for lung cancer treatments.^45^

Glioma is an intracranial cancer that is highly invasive and resistant to available drugs, making the prognosis for glioma patients very poor.^46^ Linc00152 binds to miR‐16 in human glioma cells to induce proliferation, migration, and invasion, which can be further enhanced by the induction of *BMI1* expression. Thus, linc00152 can be considered as an oncogene influencing cancer progression. A previous study on glioma stem cells (ie a glioma subtype) by Yu at al. 47 revealed that the overexpression of linc00152 can accelerate cell proliferation, migration, and invasion, but inhibit apoptosis because of an interaction with a tumor suppressor, miR‐103a‐3p. Furthermore, they also reported that linc00152 can increase the production of embryonic zinc finger protein 1 (FEZF1), which then targets miR‐103a‐3p and facilitates promoter activities that initiate the expression of the oncogenic gene encoding cell division cycle 25A (CDC25A), which further activates PI3K/AKT pathways that mediate the malignant behavior of glioma stem cells.^48^

In the hepatitis B virus‐associated HCC, the effects of linc00152 on tumor progression are the result of increased linc00152 transcription due to the X protein (HBx). The suppression of linc00152 inhibits cell proliferation and invasion.^49^ A different mechanism is also responsible for the development of HCC, during which linc00152 targets the *EpCAM* promoter to stimulate the mTOR pathway to promote cell proliferation.^20^

The linc00152 expression level is significantly higher in clear cell renal cell carcinoma tissues than in the paired adjacent normal tissues, and the upregulated linc00152 expression is correlated with TNM stage. A previously conducted multivariate analysis illustrated that linc00152 can be used as an independent prognostic factor in patients with clear cell renal cell carcinoma, with high linc00152 expression levels associated with a poor prognosis.^34^ Downregulated linc00152 expression delays the proliferation and invasion of clear cell renal carcinoma cells, while apoptosis is accelerated, indicating that linc00152 affects the occurrence and development of clear cell renal cell carcinoma. However, the mechanism underlying the linc00152‐mediated regulation of tumor cell proliferation and invasion in clear cell renal carcinoma will need to be characterized in future studies.^34^

Furthermore, other mechanisms exist in different cancer types. For example, linc00152 is deregulated during breast cancer progression.^21^ Triple‐negative breast cancer is a type of basal‐like breast cancer, whose development is related to the suppressive effect of linc00152 on BRCA1/PTEN via mammalian DNA methyltransferase.^36^

In colorectal cancer, linc00152 promotes the proliferation, migration, and invasion in the miR‐139‐5p/NOTCH 1 axis, while also inducing the resistance of colorectal cancer cells to 5‐fluorouracil‐mediated apoptosis.^50^, ^51^ Moreover, linc00152 can facilitate the epithelial‐to‐mesenchymal transition and metastasis in colon cancer by binding to cytoplasmic β‐catenin, thereby impeding casein kinase 1 (CK1)‐induced β‐catenin phosphorylation and enabling β‐catenin as well as linc00152 to be translocated into the nucleus in a positive feed‐forward circuit.^52^

The progression of renal cell carcinoma is correlated with the elevated level of linc00152, which contributes to lymph node metastases, advances in TNM stage, and shorter overall survival. Additionally, downregulated linc00152 expression decreases cell proliferation and shortens the S phase, possibly because of the epigenetic suppression of P16 and inhibition of miR‐205. An earlier investigation involving RNA immunoprecipitation and chromatin immunoprecipitation confirmed that linc00152 binds to EZH2, lysine‐specific histone demethylase 1 (LSD1), and histone H3 during the pathogenesis of renal cell carcinoma.^53^

Nötzold et al 54 detected upregulated linc00152 expression in lung, liver, and breast cancers, but not in Burkitt's lymphoma. A cell viability assay indicated that inhibited linc00152 expression can disrupt cell proliferation. They also demonstrated that the knockdown of linc00152 by a prometaphase arrest can indirectly induce the apoptosis of transfected HeLa cells, further implying that linc00152 may participate in the cell cycle by interacting with proteins related to the M phase; however, the mechanism mediating this interaction remains unclear.^54^ Other studies have also suggested that linc00152 affects the cell cycle.^27^, ^29^, ^34^, ^36^, ^54^

### 3.2 Association of linc00152 with tumor size and pathological grades

Li et al 55 revealed the significant relationship between plasma linc00152 expression and tumor size and stage in NSCLC. Moreover, the linc00152 expression level in lung cancer is correlated with tumor volume, lymph node metastases, and patient survival.^45^, ^56^

**Figure 1:**
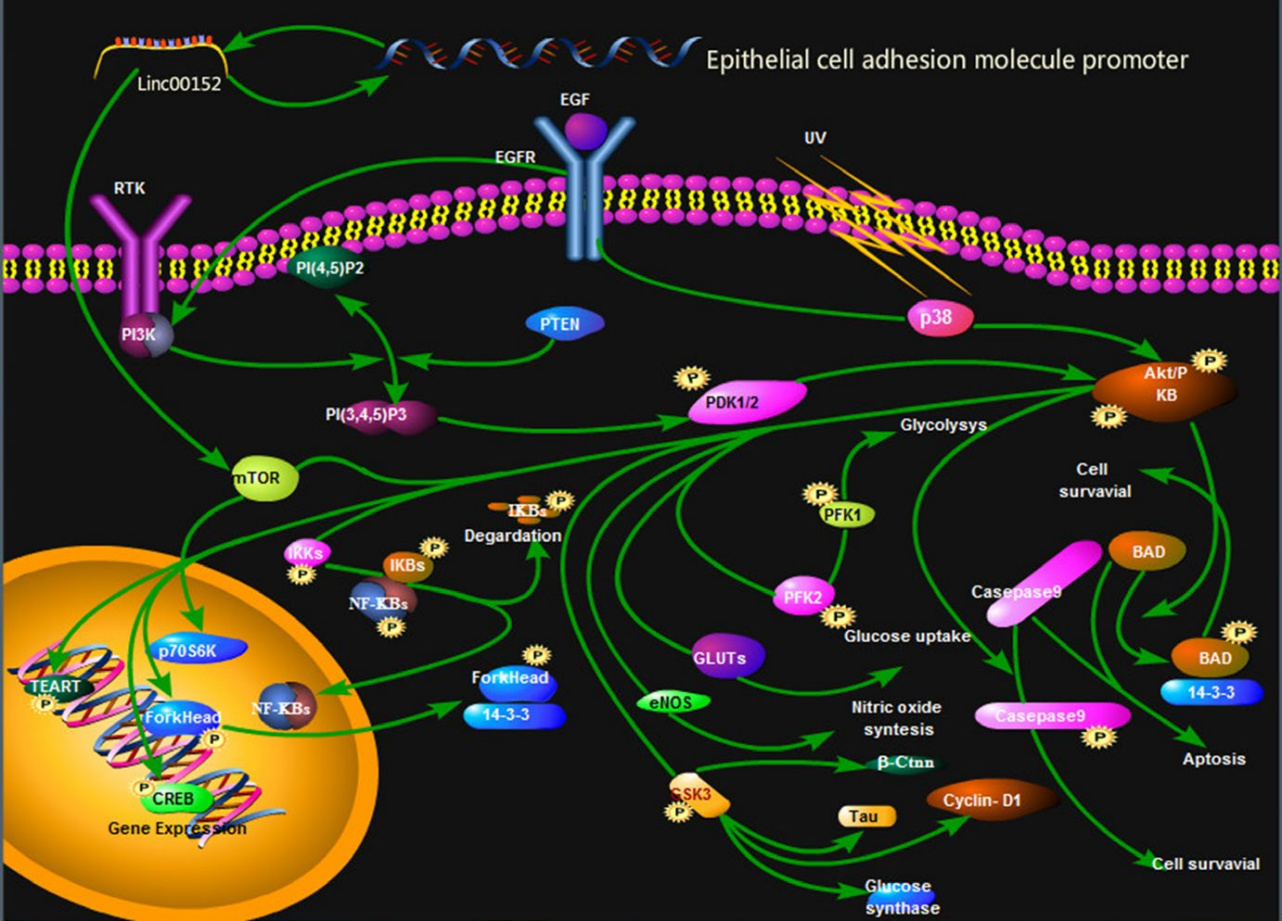
Linc00152 promotes the proliferation of HCC cells by activating the mTOR signaling pathway

Ji et al 20 reported that linc00152 is overexpressed in HCC tissues and cell lines, and its expression is associated with tumor size and the Edmondson pathological grade, but it has little influence on tumor cell invasion and apoptosis. A functional gain and deletion model analysis indicated that linc00152 overexpression in vitro and in vivo can promote the proliferation of HCC cells and the growth of a xenograft tumor in mice. The associated mechanism may be related to the activation of the mTOR signaling pathway (Figure 1) followed by an interaction with an epithelial cell adhesion molecule promoter. The average linc00152 expression level in gastric cancer is also associated with tumor size, but not with the metastasis or differentiation.^28^, ^57^ These results suggest that linc00152 is important for regulating the progress of liver cancer, and may be clinically useful for the diagnosis and treatment of liver cancer.^49^

### 3.3 Dual role of linc00152 as an oncogene and tumor‐suppressor gene

**Figure 2:**
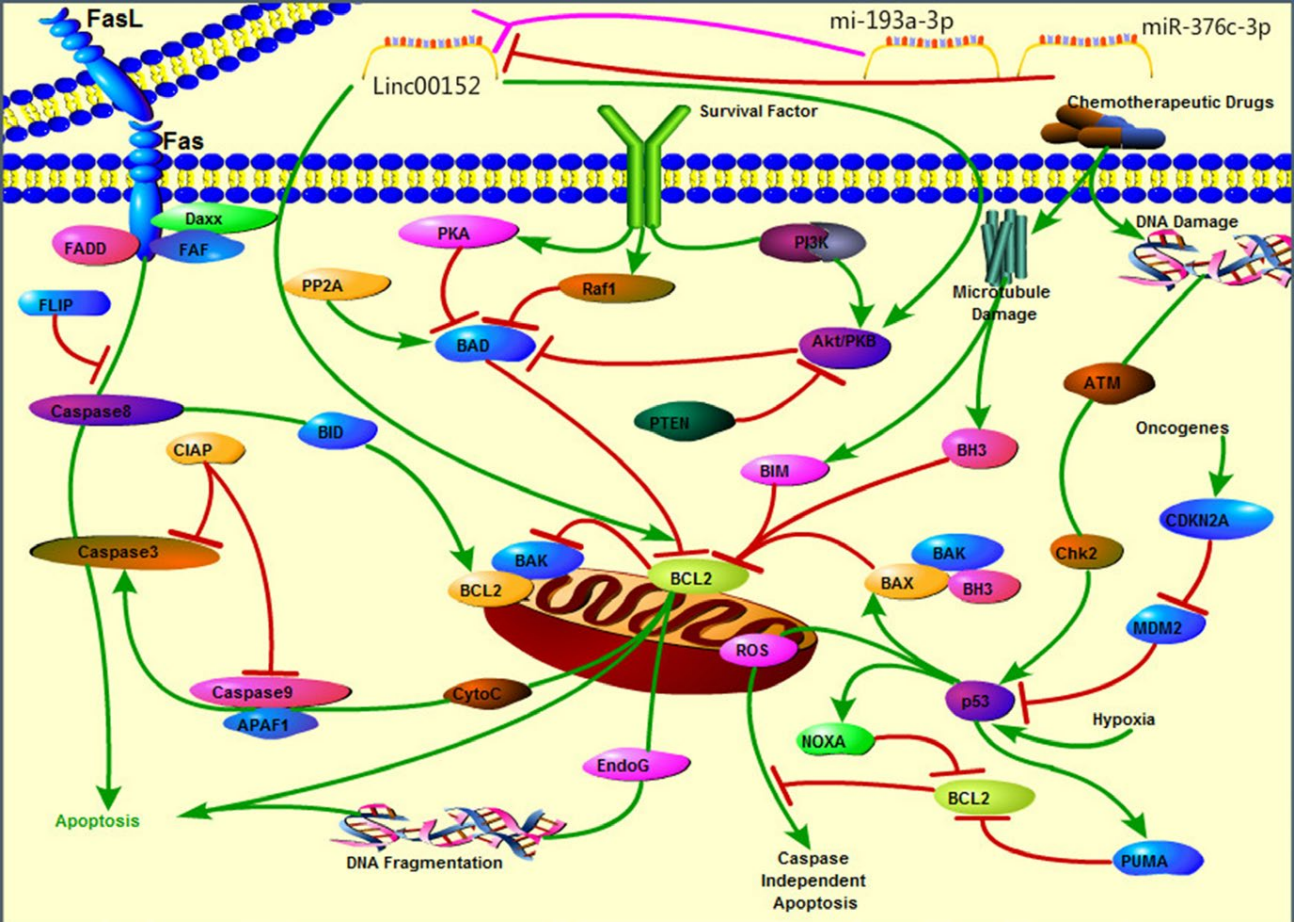
Linc00152 has a dual role as an oncogene and tumor‐suppressor gene in colon cancer

On the basis of several studies, linc00152 represents an oncogene in diverse cancers,^48^, ^58^ including gastric cancer,^28^, ^29^, ^31^, ^32^ liver cancer,^20^ HCC, colon cancer, gallbladder cancer,^39^, ^41^ and renal cell carcinoma.^34^ Zhang et al 12 reported that linc00152 expression is lower in colon cancer tissues and cell lines than in normal tissues and cells. Additionally, transfecting colon cancer cells with the linc00152 gene decreases cellular activity, but enhances apoptosis.^37^ In‐depth research revealed that the hypoxic microenvironment of cancer tissues can increase the abundance of miR‐376c‐3p, which can negatively regulate linc00152 expression, and affect its target genes (eg, *ki‐67*, *Bcl‐2*, and *Fas*) to activate apoptosis pathways. Thus, linc00152 may have a dual role as an oncogene and tumor‐suppressor gene in colon cancer^38^, ^59^ (Figure 2).

## 4 REGULATION OF LINC00152 IN HUMAN TUMORS

**Figure 3:**
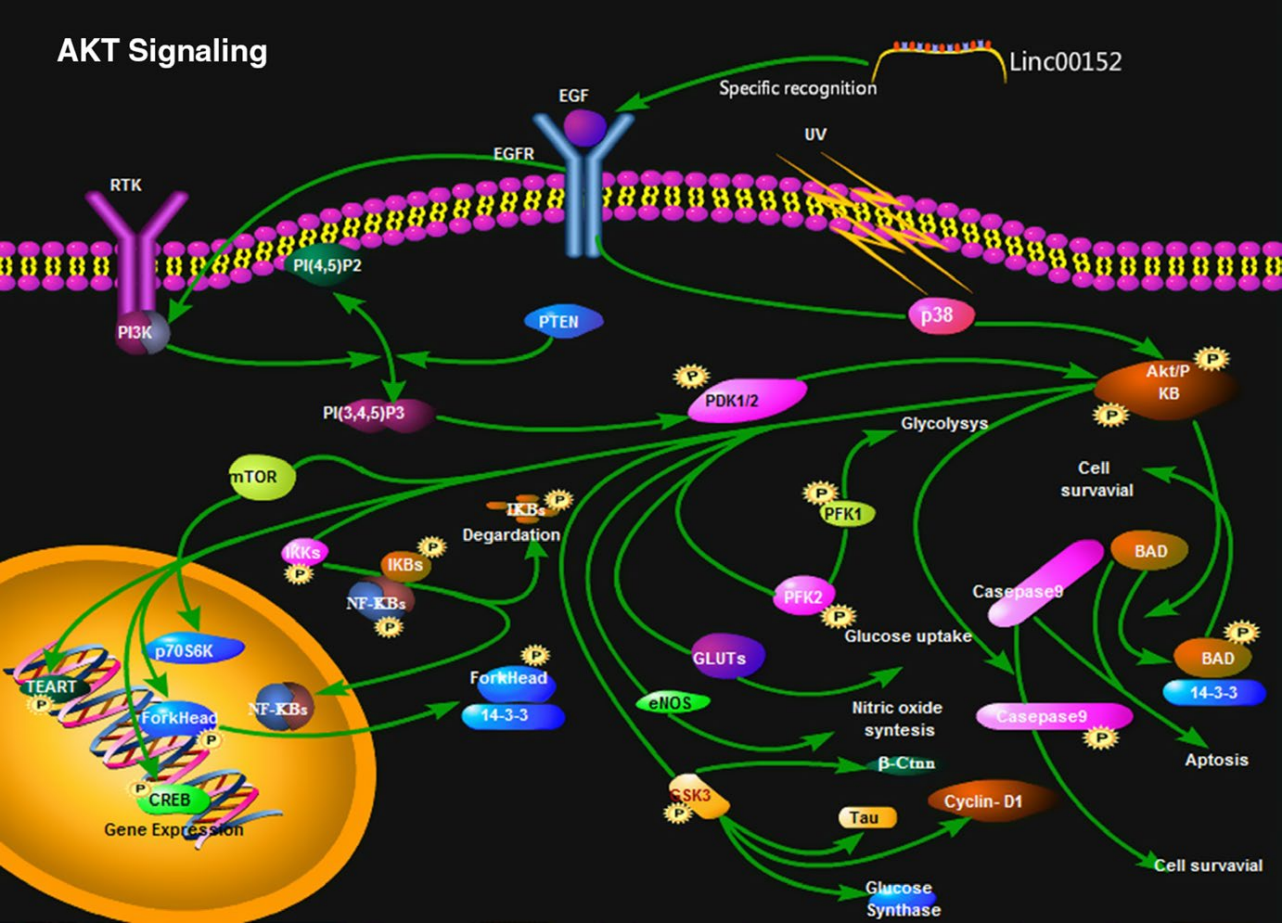
Linc00152 promotes the proliferation of gastric cancer cells by regulating the PI3K/AKT signaling pathway

When we investigated the relationship between linc00152 and gastric carcinogenesis, we observed that linc00152 can specifically recognize the EGFR‐binding site and activate the PI3K/AKT signaling pathway to promote the proliferation of gastric cancer cells.^31^ Moreover, PI3K signals help regulate many cellular functions such as proliferation, differentiation, apoptosis, and glucose transport. The signaling pathway comprising type IA PI3K and its downstream molecular protein kinase B (PKB or AKT) was recently determined to be closely related to the development and progression of human tumors.^32^ This pathway regulates the proliferation and survival of tumor cells, and its abnormal activity can lead to the development of malignant cells, while also facilitating cellular migration, adhesion, tumor angiogenesis, and the degradation of the extracellular matrix (Figure 3). Furthermore, p15 and p21 are important tumor‐suppressor factors regulating the cell cycle. Linc00152 can downregulate the expression of *p15* and *p21*, leading to an unregulated cell cycle.^29^ Thus, linc00152 influences the occurrence and development of gastric cancer by regulating cell proliferation and signaling pathways related to the cell cycle.

Linc00152 is more highly expressed in colon cancer tissues than in normal tissues, and this upregulated expression is positively correlated with the clinical stage and lymph node metastases.^41^ Functional analyses indicated that linc00152 can promote cell proliferation and chemotherapeutic drug resistance. The mechanism responsible for these observations may be related to linc00152 functioning as a ceRNA that binds to miR‐193a‐3p, which can upregulate the expression of *EGFR4*, and then activates the AKT signaling pathway, eventually increasing the drug resistance of tumor cells.^35^

Yang et al 60 observed that in esophageal cancer tissues and cells, miR‐17‐92 and miR‐15a/16‐1 are important posttranscriptional regulators, and their activities can be affected by linc00152, which in turn upregulates the expression of *EGFR* and promotes the proliferation and differentiation of tumor cells. Therefore, inhibiting linc00152 activity may be a viable strategy for treating esophageal cancer.

In addition to its molecular functions in diverse malignant tumors, linc00152 also plays a regulatory role in the development of benign tumors. Liu et al 37 conducted lncRNA microarray and high‐throughput sequencing analyses, and determined that many lncRNAs are expressed in infantile hemangiomas. Liu et al 23 also observed that lncRNAs are abnormally expressed in infantile hemangiomas, with linc00152 levels that are 7.84 times higher than those in normal blood vessel tissues. Moreover, an analysis of the gene ontology database confirmed that linc00152 overexpression is associated with angiogenesis‐related pathways. These studies suggest that linc00152 may be related to the occurrence of infant hemangiomas. Furthermore, there has recently been growing evidence that the mutual regulation of lncRNAs, miRNAs, and the downstream target genes is closely related to the occurrence and development of tumors.^61^

## 5 CLINICAL APPLICATION OF LINC00152 IN CANCERS

### 5.1 High linc00152 expression levels are associated with shorter survival and poor prognosis

The poor prognosis and very short overall survival of many intractable cancers are strictly related to linc0052 overexpression, including esophageal squamous cell carcinoma,^60^ lung cancer,^45^, ^56^ and hepatitis B virus‐associated HCC.^49^ Another study demonstrated that poor prognosis and chemoresistance in several cancers can be induced by the binding between EZH2 and increasing amounts of linc00152.^62^

### 5.2 Linc00152 expression may be useful for diagnosing and predicting the prognosis of cancers

High linc00152 levels have been detected in NSCLC samples, but not in benign tumors or healthy samples. Additionally, linc00152 expression may be useful for predicting the possibility of recurrence in postoperative patients. Consequently, linc00152 represents a potential biomarker for diagnosing and predicting the prognosis of cancers. Researchers have recommended considering both linc00152 and CEA to possibly improve diagnostic accuracy.^55^ Li et al 11 reported that linc00152 is overexpressed in the peripheral blood cells of HCC patients, and its expression level is closely related to tumor size, differentiation, invasion of the capsule, and the TNM stage, which influences the utility of linc0052 for diagnosing cancers. Furthermore, the combined detection of linc00152 and AFP markers in the blood can improve the diagnosis of early‐stage liver cancer.

Hu et al 60 applied bioinformatics methods to study esophageal cancer, and revealed that linc00152 expression levels are significantly higher in esophageal squamous cell carcinoma cells and dysplastic esophageal tissues than in normal esophageal tissues. Moreover, linc00152 expression is correlated with the poor prognosis of esophageal squamous cell carcinoma. Meanwhile, a risk score formula implied that linc00152 may be a potential molecular biomarker for the early prediction of esophageal squamous cell carcinoma. Linc00152 expression levels also represent a “fingerprint” for predicting HCC tumorigenesis.^62^ Because the linc00152 expression level is highly correlated with tongue squamous cell carcinoma progression, relapse, and invasion, and is responsible for the short overall survival, linc00152 can also be applied for diagnosing tongue squamous cell carcinoma and monitoring the prognosis of patients.^63^ A multivariate analysis suggested that linc00152 may be useful as an independent prognostic factor in patients with colon cancer.^64^ The potential utility of linc00152 as a biomarker has also been suggested based on earlier studies examining gastric cancer .^31^, ^32^, ^65^

Several meta‐analyses have verified the application of linc0012 as a biomarker for diagnosing cancers.^66^, ^67^ A meta‐analysis of eight trials unveiled strong correlations between linc00152 and the diagnosis, prognosis, and metastasis of cancers, particularly lymph node metastases.^68^ A recent meta‐analysis of the data for 913 cancer patients further confirmed the diagnostic utility of linc00152 as well as its association with lymph node metastases, advances in the TNM stage, and shorter overall survival.^69^ Because many studies have revealed the mechanisms underlying linc00152 effects in diverse cancers (Table 1), linc00152 or the molecules associated with its activities represent candidate therapeutic targets.

In summary, linc00152 is abnormally expressed in a variety of tumor tissues, and is closely related to the proliferation, invasion, metastasis, apoptosis, and chemoresistance of tumor cells. Using relevant technologies to disrupt linc00152 expression can affect the biological behavior of tumors, thereby providing new options for tumor diagnoses, targeted therapies, and evaluations of curative effects. However, the available research regarding linc00152 has been mostly limited to the primary functional stage of digestive system tumors. The relationships among linc00152, other types of tumors, and specific regulatory mechanisms in tumors remain unknown. It is also unclear whether linc00152 cooperatively functions with other lncRNAs or miRNAs to regulate the occurrence and development of tumors. The continuous application of new technologies and the development of related disciplines will more comprehensively characterize linc00152 activities. Furthermore, linc00152 may be useful as a new tumor molecular biomarker.

## CONFLICT OF INTEREST

There are no conflicts of interest associated with this article.

## ETHICAL APPROVAL

This article does not contain any studies with human participants or animals performed by any of the authors.

## References

[1] Ma L , Bajic VB , Zhang Z , Ma L , Bajic VB , Zhang Z . On the classification of long non‐coding RNAs On the classification of long non‐coding RNAs. RNA Biol. 2013;10(6):924‐933. 10.4161/rna.24604 PMC4111732 23696037

[2] Xiaoqing H , Dandan L , Juan W . Long non‐coding RNAs in plants. Yi chuan = Hered. 2015;37(4):344‐359. 10.16288/j.yczz.14-432 25881700

[3] Zhao W , Mu Y , Ma L , et al. Systematic identification and characterization of long intergenic non‐coding RNAs in fetal porcine skeletal muscle development. Sci Rep. 2015;5:1‐8. 10.1038/srep08957 PMC4354164 25753296

[4] Derrien T , Johnson R , Bussotti G , et al. The GENCODE v7 catalog of human long noncoding RNAs: analysis of their gene structure, evolution, and expression. Genome Res. 2012;22(9):1775‐1789. 22955988 10.1101/gr.132159.111 PMC3431493

[5] Han L , Zhang K , Shi Z , et al. LncRNA profile of glioblastoma reveals the potential role of lncRNAs in contributing to glioblastoma pathogenesis. Int J Oncol. 2012;40(6):2004‐2012. 22446686 10.3892/ijo.2012.1413

[6] Cabili MN , Trapnell C , Goff L , et al. Integrative annotation of human large intergenic noncoding RNAs reveals global properties and specific subclasses. Genes Dev. 2011;25(18):1915‐1927. 21890647 10.1101/gad.17446611 PMC3185964

[7] Liu X‐H , Sun M , Nie F‐Q , et al. Lnc RNA HOTAIR functions as a competing endogenous RNA to regulate HER2 expression by sponging miR‐331‐3p in gastric cancer. Mol Cancer. 2014;13:92. 24775712 10.1186/1476-4598-13-92 PMC4021402

[8] Wang L , Zhao YU , Bao X , et al. Dum interacts with Dnmts to regulate Dppa2 expression during myogenic differentiation and muscle regeneration. Cell Res. 2015;25(3):335‐350. 25686699 10.1038/cr.2015.21 PMC4349245

[9] Giannakakis A , Zhang J , Jenjaroenpun P , et al. Contrasting expression patterns of coding and noncoding parts of the human genome upon oxidative stress. Sci Rep. 2015;5:9737. 26024509 10.1038/srep09737 PMC4448690

[10] Kim D , Marinov G , Pepke S , et al. Single‐cell transcriptome analysis reveals dynamic changes in lncRNA expression during reprogramming. Cell Stem Cell. 2015;16(1):88‐101. 25575081 10.1016/j.stem.2014.11.005 PMC4291542

[11] Li J , Wang X , Tang J , et al. HULC and Linc00152 act as novel biomarkers in predicting diagnosis of hepatocellular carcinoma. Cell Physiol Biochem. 2015;37(2):687‐696. 26356260 10.1159/000430387

[12] Zhang Y‐H , Fu J , Zhang Z‐J , Ge C‐C , Yi Y . LncRNA‐LINC00152 down‐regulated by miR‐376c‐3p restricts viability and promotes apoptosis of colorectal cancer cells. Am J Transl Res. 2016;8(12):5286‐5297. 28078002 PMC5209482

[13] Djebali S , Davis CA , Merkel A , et al. Landscape of transcription in human cells. Nature. 2012;489(7414):101‐108. 22955620 10.1038/nature11233 PMC3684276

[14] Dinger ME , Amaral PP , Mercer TR , et al. Long noncoding RNAs in mouse embryonic stem cell pluripotency and differentiation. Genome Res. 2008;18(9):1433‐1445. 18562676 10.1101/gr.078378.108 PMC2527704

[15] Liu Y , Ferguson JF , Xue C , et al. Tissue‐specific RNA‐Seq in human evoked inflammation identifies blood and adipose LincRNA signatures of cardiometabolic diseases. Arterioscler Thromb Vasc Biol. 2014;34(4):902‐912. 24504737 10.1161/ATVBAHA.113.303123 PMC3966947

[16] Khalil AM , Guttman M , Huarte M , et al. Many human large intergenic noncoding RNAs associate with chromatin‐modifying complexes and affect gene expression. Proc Natl Acad Sci USA. 2009;106(28):11667‐11672. 19571010 10.1073/pnas.0904715106 PMC2704857

[17] Hwangbo W , Lee JH , Ahn S , et al. EGFR gene amplification and protein expression in invasive ductal carcinoma of the breast. Korean J Pathol. 2013;47(2):107‐115. 23667369 10.4132/KoreanJPathol.2013.47.2.107 PMC3647122

[18] Kalman B , Szep E , Garzuly F , Post DE . Epidermal growth factor receptor as a therapeutic target in glioblastoma. Neuromol Med. 2013;15(2):420‐434. 10.1007/s12017-013-8229-y 23575987

[19] Oliveira‐Cunha M , Hadfield KD , Siriwardena AK , Newman W . EGFR and KRAS mutational analysis and their correlation to survival in pancreatic and periampullary cancer. Pancreas. 2012;41(3):428‐434. 22422135 10.1097/MPA.0b013e3182327a03

[20] Ji J , Tang J , Deng L , et al. LINC00152 promotes proliferation in hepatocellular carcinoma by targeting EpCAM via the mTOR signaling pathway. Oncotarget. 2015;6(40):42813‐42824. 26540343 10.18632/oncotarget.5970 PMC4767473

[21] Wu J , Shuang Z , Zhao J , et al. Linc00152 promotes tumorigenesis by regulating DNMTs in triple‐negative breast cancer. Biomed Pharmacother. 2018;97:1275‐1281. 29156515 10.1016/j.biopha.2017.11.055

[22] Haque S‐U , Niu L , Kuhnell D , et al. Differential expression and prognostic value of long non‐coding RNA in HPV‐negative head and neck squamous cell carcinoma. Head Neck. 2018;40(7):1555‐1564.. 29575229 10.1002/hed.25136 PMC6037541

[23] Chen Q , Chen X , Chen Z , et al. Long intergenic non‐coding RNA 00152 promotes lung adenocarcinoma proliferation via interacting with EZH2 and repressing IL24 expression. Mol Cancer. 2017;16(1):1‐13. 10.1186/s12943-017-0581-3. 28109288 PMC5251237

[24] Yu X , Lin Y , Sui W , Zou Y , Lv Z . Analysis of distinct long noncoding RNA transcriptional fingerprints in pancreatic ductal adenocarcinoma. Cancer Med. 2017;6(3):673‐680. 28220683 10.1002/cam4.1027 PMC5345666

[25] Yang T , Zeng H , Chen W , et al. Helicobacter pylori infection, H19 and LINC00152 expression in serum and risk of gastric cancer in a Chinese population. Cancer Epidemiol. 2016;44:147‐153. 27592063 10.1016/j.canep.2016.08.015

[26] Cao W‐J , Wu H‐L , He B‐S , Zhang Y‐S , Zhang Z‐Y . Analysis of long non‐coding RNA expression profiles in gastric cancer. World J Gastroenterol. 2013;19(23):3658‐3664. 23801869 10.3748/wjg.v19.i23.3658 PMC3691033

[27] Zhao J , Liu Y , Zhang W , et al. Long non‐coding RNA Linc00152 is involved in cell cycle arrest, apoptosis, epithelial to mesenchymal transition, cell migration and invasion in gastric cancer. Cell Cycle. 2015;14(19):3112‐3123. 26237576 10.1080/15384101.2015.1078034 PMC4825539

[28] Zhou J , Zhi X , Wang L , et al. Linc00152 promotes proliferation in gastric cancer through the EGFR‐dependent pathway. J Exp Clin Cancer Res. 2015;34(1):1‐8. 10.1186/s13046-015-0250-6. 26538117 PMC4632266

[29] Chen W‐M , Huang M‐D , Sun D‐P , et al. Long intergenic non‐coding RNA 00152 promotes tumor cell cycle progression by binding to EZH2 and repressing p15 and p21 in gastric cancer. Oncotarget. 2016;7(9):9773‐9787. 26799422 10.18632/oncotarget.6949 PMC4891083

[30] Xia T , Liao QI , Jiang X , et al. Long noncoding RNA associated‐competing endogenous RNAs in gastric cancer. Sci Rep. 2014;4:6088. 25124853 10.1038/srep06088 PMC4133709

[31] Pang Q , Ge J , Shao Y , et al. Increased expression of long intergenic non‐coding RNA LINC00152 in gastric cancer and its clinical significance. Tumor Biol. 2014;35(6):5441‐5447. 10.1007/s13277-014-1709-3 24523021

[32] Li Q , Shao Y , Zhang X , et al. Plasma long noncoding RNA protected by exosomes as a potential stable biomarker for gastric cancer. Tumor Biol. 2015;36(3):2007‐2012. 10.1007/s13277-014-2807-y 25391424

[33] Müller S , Raulefs S , Bruns P , et al. Next‐generation sequencing reveals novel differentially regulated mRNAs, lncRNAs, miRNAs, sdRNAs and a piRNA in pancreatic cancer. Mol Cancer. 2015;14(1):94. 25910082 10.1186/s12943-015-0358-5 PMC4417536

[34] Wu Y , Tan C , Weng W‐W , et al. Long non‐coding RNA Linc00152 is a positive prognostic factor for and demonstrates malignant biological behavior in clear cell renal cell carcinoma. Am J Cancer Res. 2016;6(2):285‐299. 27186403 PMC4859660

[35] Yue B , Cai D , Liu C , Fang C , Yan D . Linc00152 functions as a competing endogenous RNA to confer oxaliplatin resistance and holds prognostic values in colon cancer. Mol Ther. 2016;24(12):2064‐2077. 27633443 10.1038/mt.2016.180 PMC5167786

[36] Van Grembergen O , Bizet M , de Bony EJ , et al. Portraying breast cancers with long noncoding RNAs. Sci Adv. 2016;2(9):e1600220. 27617288 10.1126/sciadv.1600220 PMC5010371

[37] Liu X , Lv R , Zhang L , et al. Long noncoding RNA expression profile of infantile hemangioma identified by microarray analysis. Tumour Biol. 2016;37(12):15977‐15987. 10.1007/s13277-016-5434-y 27709553

[38] Sun K , Hu P , Xu F . LINC00152/miR‐139‐5p regulates gastric cancer cell aerobic glycolysis by targeting PRKAA1. Biomed Pharmacother. 2018;97:1296‐1302. 29156518 10.1016/j.biopha.2017.11.015

[39] Cai Q , Wang Z‐Q , Wang S‐H , et al. Upregulation of long non‐coding RNA LINC00152 by SP1 contributes to gallbladder cancer cell growth and tumor metastasis via PI3K/AKT pathway. Am J Transl Res. 2016;8(10):4068‐4081. 27829993 PMC5095302

[40] Cai Q , Wang Z , Wang S , et al. Long non‐coding RNA LINC00152 promotes gallbladder cancer metastasis and epithelial‐mesenchymal transition by regulating HIF‐1alpha via miR‐138. Open Biol. 2017;7(1):160247. 28077595 10.1098/rsob.160247 PMC5303272

[41] Zhang Y , Xiang C , Wang Y , et al. lncRNA LINC00152 knockdown had effects to suppress biological activity of lung cancer via EGFR/PI3K/AKT pathway. Biomed Pharmacother. 2017;94:644‐651. 28787699 10.1016/j.biopha.2017.07.120

[42] Chen J , Xia D , Luo JD , Wang P . Exogenous p27KIP1 expression induces anti‐tumour effects and inhibits the EGFR/PI3K/Akt signalling pathway in PC3 cells. Asian J Androl. 2009;11(6):669‐677. 19734935 10.1038/aja.2009.51 PMC3735325

[43] Collisson EA , Campbell JD , Brooks AN , et al. Comprehensive molecular profiling of lung adenocarcinoma. Nature [Internet]. 2014;511(7511):543‐550. Available from http://www.nature.com/doifinder/10.1038/nature13385. 25079552 10.1038/nature13385 PMC4231481

[44] Okayama H , Kohno T , Ishii Y , et al. Identification of genes upregulated in ALK‐positive and EGFR/KRAS/ALK‐negative lung adenocarcinomas. Cancer Res. 2012;72(1):100‐111. 22080568 10.1158/0008-5472.CAN-11-1403

[45] Feng S , Zhang J , Su W , et al. Overexpression of LINC00152 correlates with poor patient survival and knockdown impairs cell proliferation in lung cancer. Sci Rep. 2017;7(1):2982. 28592840 10.1038/s41598-017-03043-x PMC5462773

[46] Zheng J , Liu X , Xue Y , et al. TTBK2 circular RNA promotes glioma malignancy by regulating miR‐217/HNF1beta/Derlin‐1 pathway. J Hematol Oncol. 2017;10:52. 28219405 10.1186/s13045-017-0422-2 PMC5319142

[47] Chen X , Li D , Gao Y , et al. Long intergenic noncoding RNA 00152 promotes glioma cell proliferation and invasion by interacting with MiR‐16. Cell Physiol Biochem. 2018;46(3):1055‐1064. 10.1159/000488836. Epub 2018 Apr 13. 29669323

[48] Yu M , Xue Y , Zheng J , et al. Linc00152 promotes malignant progression of glioma stem cells by regulating miR‐103a‐3p/FEZF1/CDC25A pathway. Mol Cancer. 2017;16(1):110 10.1186/s12943-017-0677-9. 28651608 PMC5485714

[49] Deng X , Zhao XF , Liang XQ , Chen R , Pan YF , Liang J . Linc00152 promotes cancer progression in hepatitis B virus‐associated hepatocellular carcinoma. Biomed Pharmacother. 2017;90:100‐108. 28343069 10.1016/j.biopha.2017.03.031

[50] Bian Z , Zhang J , Li M , et al. Long non‐coding RNA LINC00152 promotes cell proliferation, metastasis, and confers 5‐FU resistance in colorectal cancer by inhibiting miR‐139‐5p. Oncogenesis. 2017;6(11):395. 29180678 10.1038/s41389-017-0008-4 PMC5868057

[51] Yue B , Cai D , Liu C , Fang C , Yan D . Linc00152 functions as acompeting endogenous rna to confer oxaliplatin resistance and holds prognostic valuesn in colon cancer. Mol Ther. 2016;24:2064‐2077. 27633443 10.1038/mt.2016.180 PMC5167786

[52] Yue B , Liu C , Sun H , et al. A positive feed‐forward loop between LncRNA‐CYTOR and Wnt/β‐Catenin signaling promotes metastasis of colon cancer. Mol Ther. 2018;26(5):1287‐1298. 29606502 10.1016/j.ymthe.2018.02.024 PMC5993983

[53] Wang Y , Liu J , Bai H , Dang Y , Lv P , Wu S . Long intergenic non‐coding RNA 00152 promotes renal cell carcinoma progression by epigenetically suppressing P16 and negatively regulates miR‐205. Am J Cancer Res. 2017;7(2):312‐322. 28337379 PMC5336504

[54] Nötzold L , Frank L , Gandhi M , et al. The long non‐coding RNA LINC00152 is essential for cell cycle progression through mitosis in HeLa cells. Sci Rep. 2017;7(1):2265. 28536419 10.1038/s41598-017-02357-0 PMC5442156

[55] Li N , Feng XB , Tan Q , et al. Identification of circulating long noncoding RNA Linc00152 as a novel biomarker for diagnosis and monitoring of non‐small‐cell lung cancer. Dis Markers. 2017;2017:1‐8. 10.1155/2017/7439698 PMC5742528 29375177

[56] Zhang P , Wang Y , Weng W , et al. Linc00152 promotes cancer cell proliferation and invasion and predicts poor prognosis in lung adenocarcinoma. J Cancer. 2017;8(11):2042‐2050. 28819405 10.7150/jca.18852 PMC5559966

[57] Zhou J , Zhi X , Wang L , et al. Erratum to: Linc00152 promotes proliferation in gastric cancer through the EGFR‐dependent pathway. J Exp Clin Cancer Res. 2016;9(35):30 10.1186/s13046-015-0262-2. PMC4748527 26860954

[58] Yu Y , Yang J , Li Q , Xu B , Lian Y , Miao L . LINC00152: a pivotal oncogenic long non‐coding RNA in human cancers. Cell Prolif. 2017;;50(4):e12349. 10.1111/cpr.12349 PMC6529135 28464433

[59] Huang Y , Luo H , Li F , et al. LINC00152 down‐regulated miR‐193a‐3p to enhance MCL1 expression and promote gastric cancer cells proliferation. Biosci Rep. 2018;38(3):BSR20171607. 29339419 10.1042/BSR20171607 PMC5938421

[60] Yang S , Ning Q , Zhang G , Sun H , Wang Z , Li Y . Construction of differential mRNA‐lncRNA crosstalk networks based on ceRNA hypothesis uncover key roles of lncRNAs implicated in esophageal squamous cell carcinoma. Oncotarget. 2016;7(52):85728‐85740. 27966444 10.18632/oncotarget.13828 PMC5349869

[61] Hu H‐B , Jie H‐Y , Zheng X‐X . Three circulating LncRNA predict early progress of esophageal squamous cell carcinoma. Cell Physiol Biochem. 2016;40(1–2):117‐125. 27855375 10.1159/000452529

[62] Yuan W , Sun Y , Liu L , Zhou B , Wang S , Gu D . Circulating LncRNAs serve as diagnostic markers for hepatocellular carcinoma. Cell Physiol Biochem. 2017;44(1):125–132. 29130980 10.1159/000484589

[63] Yu J , Liu Y , Guo C , et al. Upregulated long non‐coding RNA LINC00152 expression is associated with progression and poor prognosis of tongue squamous cell carcinoma. J Cancer. 2017;8(4):523–530. 28367232 10.7150/jca.17510 PMC5370496

[64] Chen Z , Cai X , Chang LI , et al. LINC00152 is a potential biomarker involved in the modulation of biological characteristics of residual colorectal cancer cells following chemoradiotherapy. Oncol Lett. 2018;15(4):4177–4184. 29541183 10.3892/ol.2018.7833 PMC5835918

[65] Sun W , Yang Y , Xu C , Xie Y , Guo J . Roles of long noncoding RNAs in gastric cancer and their clinical applications. J Cancer Res Clin Oncol. 2016;142(11):2231–2237. 10.1007/s00432-016-2183-7. Epub 2016 May 31. Review. 27246953 PMC11819183

[66] Liu L , Wen J , Gu X , Wu D , Lu M , Zhao Q . Prognostic role of long non‐coding RNA LINC00152 in Chinese cancer patients: a meta‐analysis. Oncotarget. 2017;8(54):93227–93235. 10.18632/oncotarget.21838. eCollection. 29190992 PMC5696258

[67] Quan F‐Y , Jiang J , Zhai Y‐F , Li B , Wu X‐H , Nie W . The prognostic effect of LINC00152 for cancer: a meta‐analysis. Oncotarget. 2017;8(43):75427–75433. 29088878 10.18632/oncotarget.20130 PMC5650433

[68] Miao C , Zhao K , Zhu J , et al. Clinicopathological and prognostic role of long noncoding RNA Linc00152 in various human neoplasms: evidence from meta‐analysis. Biomed Res Int. 2017;2017:6010721 10.1155/2017/6010721. Epub 2017 Nov 23. 29285514 PMC5733223

[69] Zhang J , Yin M , Huang J , et al. Long noncoding RNA LINC00152 as a novel predictor of lymph node metastasis and survival in human cancer: a systematic review and meta‐analysis. Clin Chim Acta. 2018;483:25–32. 10.1016/j.cca.2018.03.034. [Epub ahead of print]. 29617624

